# Going beyond PA: Assessing sensorimotor capacity with wearables in multiple sclerosis—a cross-sectional study

**DOI:** 10.1186/s12984-023-01247-z

**Published:** 2023-09-21

**Authors:** Philipp Gulde, Heike Vojta, Stephanie Schmidle, Peter Rieckmann, Joachim Hermsdörfer

**Affiliations:** 1 Chair of Human Movement Science, Department of Sport and Health Sciences, Technical University of Munich, Munich, Germany; 2 Centre for Clinical Neuroplasticity, Medical Park Loipl, Medical Park SE, Bischofswiesen, Germany; 3 Friedrich-Alexander University Erlangen-Nurnberg, Erlangen, Germany

**Keywords:** Multiple sclerosis, Physical activity, Sensorimotor capacity, Accelerometer, Gyroscope, Wrist-worn actigraphy, Smartwatch

## Abstract

**Background:**

Wearable technologies are currently clinically used to assess energy expenditure in a variety of populations, e.g., persons with multiple sclerosis or frail elderly. To date, going beyond physical activity, deriving sensorimotor capacity instead of energy expenditure, is still lacking proof of feasibility.

**Methods:**

In this study, we read out sensors (accelerometer and gyroscope) of smartwatches in a sample of 90 persons with multiple sclerosis over the course of one day of everyday life in an inpatient setting. We derived a variety of different kinematic parameters, in addition to lab-based tests of sensorimotor performance, to examine their interrelation by principal component, cluster, and regression analyses.

**Results:**

These analyses revealed three components of behavior and sensorimotor capacity, namely clinical characteristics with an emphasis on gait, gait-related physical activity, and upper-limb related physical activity. Further, we were able to derive four clusters with different behavioral/capacity patterns in these dimensions. In a last step, regression analyses revealed that three selected smartwatch derived kinematic parameters were able to partially predict sensorimotor capacity, e.g., grip strength and upper-limb tapping.

**Conclusions:**

Our analyses revealed that physical activity can significantly differ between persons with comparable clinical characteristics and that assessments of physical activity solely relying on gait can be misleading. Further, we were able to extract parameters that partially go beyond physical activity, with the potential to be used to monitor the course of disease progression and rehabilitation, or to early identify persons at risk or a sub-clinical threshold of disease severity.

## Background

To date, a variety of different wearable technologies exist to assess physical activity (PA), using time series of heart rate, acceleration signals, and acceleration derived pedometer counts [2, 4, 17, 37]. The application has a broad scope, covering the complete age range from juveniles to elderly and a comprehensive spectrum of health states, i.e., healthy populations with the aim to foster a healthy lifestyle or supervise training loads in athletes, as well as clinical populations, like frail elderly, stroke survivors, or persons with neurodegenerative diseases, for instance Parkinson’s disease or multiple sclerosis (MS) [4, 5, 7, 19, 28, 31, 33]. Currently, the main target is to make a rough estimate of energy expenditure, whether by the number of daily steps, the intensity of movements, or increases from resting heart rate. The predictive value of such data is high [3, 17, 37, 38]. To date and to the best of our knowledge, however, none of these technologies, or better: none of the current derived parameters, are capable to go beyond pure energy expenditure and provide estimates of the wearer’s (health-related) sensorimotor capacity, although there have been first attempts [7, 21, 23]. For instance, comparing a healthy person with sedentary behavior with a neurologically impaired person who pursues a highly active lifestyle by means of the PA levels could lead to wrong assumptions about the course of the disease and prevent early detection or knowledge on therapeutic success (e.g., by drug or training interventions). While PA can be a predictor, or risk factor, as well as the consequence of disease [1, 3, 6, 22, 38], many (neuro)degenerative diseases and syndromes show early and strong sensorimotor impairments. This particularly, highlights the importance of, additionally to energy expenditure, assessing sensorimotor capacity, which can be observed in terms of particular characteristics such as reduced movement speeds, movement smoothness, or more monotonous activity patterns [7, 12, 25, 32]. Consequently, the question is not only “How much are you moving?”, but also “How well are you moving?”. Therefore, our target was to explore the capacity of wearable technology derived data to estimate the sensorimotor capacity—to go beyond PA.

In this exploratory study, we assessed the uncontrolled (i.e., outside the lab) behavior of 90 persons with multiple sclerosis (pwMS) in an inpatient neurorehabilitation setting by the use smartwatch technology to collect data from the built-in pedometer, accelerometer, and gyroscope. MS was used as a model due to its progressive nature, a broad spectrum of potential impairments ranging from spasticity to cognitive symptoms, and its established measures of disease severity—the expanded disability status scale (EDSS), as well as its sensorimotor-based pendant—the Watzmann severity scale (WSS) [18]. By a set of different parameters, which were partially derived from previous kinematic analyses of sensorimotor performance in complex (instrumented) activities of daily living (ADL) [12, 16, 32], we were aiming to explore the potential to estimate the sensorimotor capacity in a wide range of disease severity.

## Methods

### Sample

A convenience sample of 90 patients was recruited at the Centre for Clinical Neuroplasticity, Medical Park Loipl, a specialist clinic for neurology and rehabilitation in Germany (Table 1). Inclusion criteria were a diagnosed MS (according to the patient file) and the willingness to participate in the study. Diagnoses were, dependent on the date of diagnosis, based on different criteria, which were McDonald (2017) in 23% (n = 21), McDonald (2010) in 30% (n = 27), McDonald (2005) in 13% (n = 12), McDonald (2001) in 12% (n = 11), Poser in 19% (n = 17), and Schumacher in 2% (n = 2) of the cases. None of the initial diagnoses have been revised. In that sense, we assumed a clean sample of persons with multiple sclerosis. Exclusion criteria were the presence of neurological comorbidities like, for instance, stroke. Further, due to the nature of the rehabilitation facility, a certain preselection of potential participants was unavoidable, excluding participants with EDSS scores higher or equal than 8.5, intense need for nursing, and psychiatric disorders. Ethical approval was given by the ethics committee of the Medical Faculty of the Technical University of Munich (approval identifier: 478/19 S-SR). All participants gave written informed consent to participate in the study.

**Table 1:** Sample characteristics

Dataset	Age in [a]	Biological sex	Type of MS	Time in [a] since first manifestation	EDSS	WSS
Full n = 90	50.2 ± 10.6 (25–73)	67% female 33% male	64% relapsing remitting 36% progressive (primary or secondary)	17 ± 12 (0–51)	4.0 ± 1.9 (1.0–8.0)	3.7 ± 1.6 (0.7–7.9)
PCA/Clustering n = 76	49.5 ± 10.1 (25–71)	67% female 33% male	71% relapsing remitting 29% progressive (primary of secondary)	16 ± 12 (0–49)	3.6 ± 1.7 (1.0–7.5)	3.4 ± 1.2 (0.7–5.8)

EDSS: Expanded disability status scale, WSS: Watzmann Severity Scale [18]

### Procedure

Participants underwent a sensorimotor test battery at the clinic’s Neuro Assessment Lab (NAL), which included a set of tests of upper-limb sensorimotor capacity and gait performance. On the same or following day, participants were equipped on the dominant or better functioning (i.e., predominantly used in everyday life) upper-limb with a Huawei gt2 smartwatch (Huawei Investment & Holding Co., Ltd., Shenzhen, China) for one day. The smartwatch comprised a custom Android application (**TUM:W**—standing for *Technical University of Munich Watch*) that has been developed using C# Xamarin (Visual Studio 2019; Microsoft Corp., Redmond, WA). Participants wore the watch for one full (working/therapy) day between 08:00 and 18:00. Amount and content of therapies was not controlled for. Participants were told that the watch aims to assess the quality of everyday movements rather than PA (as part of the informed consent). As visible in Table 1, not all participants had full datasets, which was in eight cases due to an inability to carry out balance and gait tests in higher EDSS grades. One participant (1 out of 1) with an EDSS of 8.0 was not able to carry out neither the balance nor the gait tests. Two participants (2 out of 4) with an EDSS of 7.5 were not able to execute the balance tasks. One participant (1 out of 2) with an EDSS of 7.0 was not able to execute neither the balance nor the gait tests and a further one (1 out of 2) was not able to carry out the balance task (total 2 out of 2). Three participants with an EDSS of 6.5 (3 out of 6) were not able to execute all gait tests.

### Parameters: patient characteristics

We recorded the following key characteristics: The age (in [a]; **AGE**), the time since first manifestation of MS (in [a]; **FM**), and the **EDSS**.

### Parameters: neuro assessment lab (NAL)

All tests of the Neuro Assessment Lab (NAL) were used to estimate the sensorimotor capacity of the participants.

**Upper-limbs:** We recorded the summed performance of dominant and non-dominant upper-limb for the nine hole peg test (due to a non-normal distribution we used the reciprocal 1/ sum of trial durations which lead to [1/s], **PEG**), a grip strength assessment using a isometric dynamometer (sex-specific z-scores of the summed force equivalent in [kg], **GRIP**), a upper-limb tapping task (sum of frequencies over the course of 10s in [Hz], **TAP**), and a pursuit task (sum of deviations from target in [mm], **DOT**).

**Information processing capacity:** Simple and go/no go reaction times (mean of 10 (go) trials per task in [ms], **RT** and **GNG**) were checked to coarsely estimate the information processing capacity of our subjects. Participants were asked to use their better functioning upper-limb to execute the tasks.

**Postural control and gait:** Further, body sway in parallel stance with open eyes and the Romberg index in a closed eyes condition (sway closed eyes/ sway open eyes) (10s trial durations, sway as the mean acceleration of the frontal part of the hip in [mm/s^2^]; **SWAY** and **ROMBERG**), and the time to execute the timed stand up and go (with 2 × 3m walking distance], in [s]; **TSUG**), the time to walk 10m at normal and maximum pace (in [s]; **NORM10** and **MAX10**) were assessed. Lastly, in gait at self-selected (“normal”) pace, the step frequency (in [Hz]; **FREQ**), the smoothness of gait (in % with 100% being perfect; **COMPLEXITY**), the signal to noise ratio of gait (in % with 100% being perfect; **NOISE**), an gait asymmetry index (ratio of second highest and highest weighting of the frequency spectrum of the acceleration signal at the sternum in % with 0% being perfect; **ASYM**), and the limping (relative deviation of acceleration maxima between the two legs during gait in % with 0% being perfect, **LIMP**) were assessed. An upper- and lower-limb laterality index (**LAT**) was computed as the relative interlimb differences of the following tests: nine hole peg test, grip strength, tapping, smooth pursuit, and limping during gait. Lastly, the **WSS** was determined on the basis of sensorimotor performance. All tests, the underlying algorithms and methodologies, as well as the validation of the WSS have been previously described in Gulde [18].

### Parameters: smartwatch (TUM:W)

All parameters of the smartwatch (TUM:W) were used to assess the level of physical activity and explore the potential of a set of mostly gyroscope-derived parameters to estimate the sensorimotor capacity that has been examined in the NAL (see above).

**Fig. 1:**
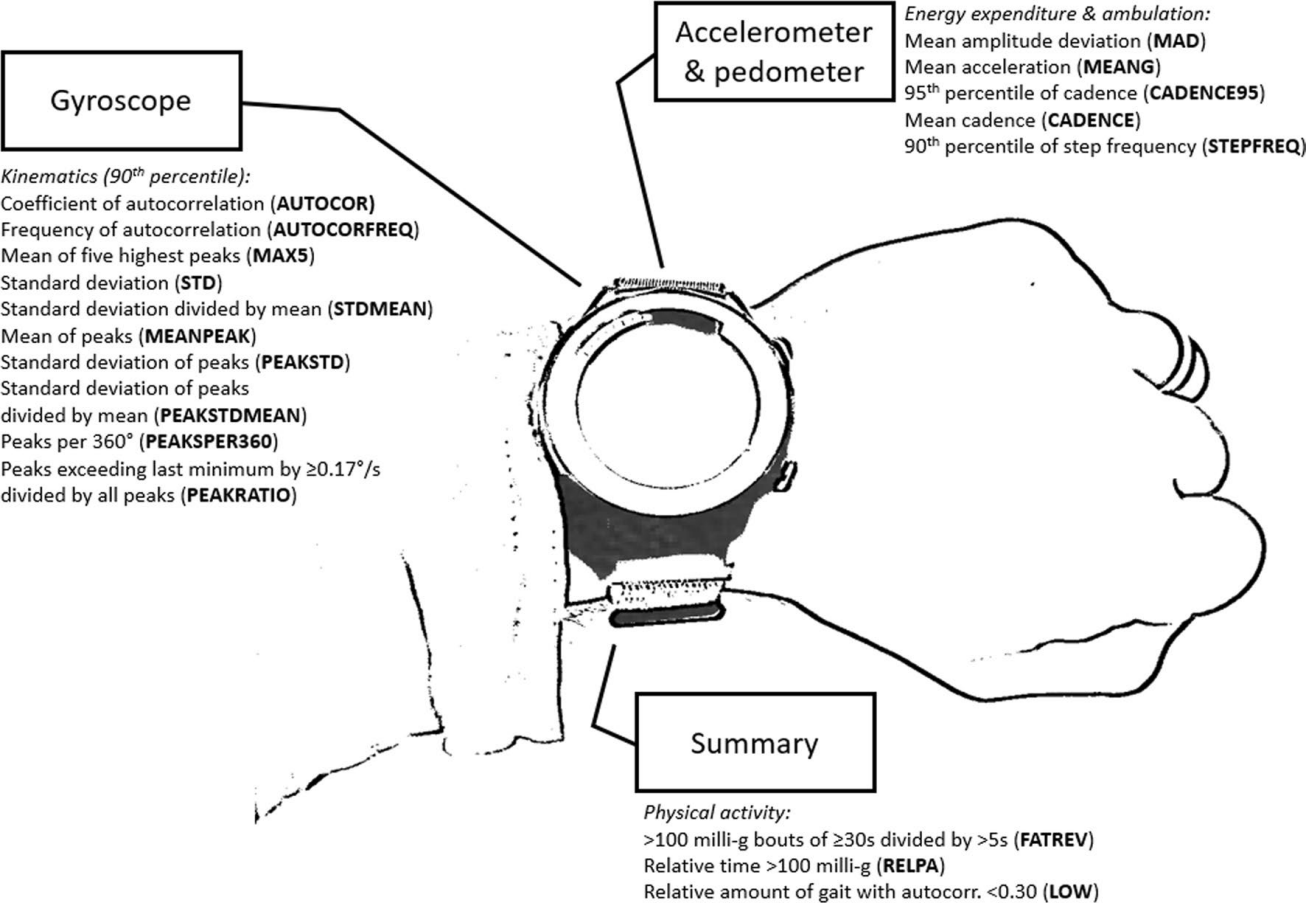
Illustration of TUM:W and its derived parameters

The measurement frequency of the accelerometer was 100Hz and of the gyroscope 25Hz. The built-in pedometer was accelerometer-based. The gyroscope data was used for kinematic analyses (spatiotemporal characteristics of behavior), so we smoothed it using a moving average of 0.42s [14]. All data processing to derive the used parameters was executed on the smartwatch to minimize needed storage. Figure 1 is an illustration of TUM:W and its derived parameters (additionally summed up and explained in Table 2).

**Table 2:** Summary of parameters derived from the TUM:W smart-watch

Abbreviation	Sensor (dimension)	Assessment	Operationalization	Outcome	(thought) Target
CADENCE95	Pedometer (PA)	60s every 60s	Steps/min	95^th^ perc	Continuous walking capacity (to cover distance)
CADENCE	Pedometer (PA)	60s every 60s	Steps/min if > 5 recognized steps	Mean	Walking fragmentation
MEANG	Accelerometer (PA)	5s every 5s	Mean acceleration	90th perc	Energy expenditure
MAD	Accelerometer (PA)	5s every 5s	Average deviation from mean of signal	90th perc	Energy expenditure
STEPFREQ	Pedometer (PA)	5s every 5s	Steps/s	90th perc	Short distance walking (e.g., indoors)
ACTI	Gyroscope (beyond PA)	5s every 5s	Mean angular vel	90th perc	Intensity of upper-limb use (e.g., during ADL)
AUTOCOR	Gyroscope (beyond PA)	5s every 2.5s	Highest coefficient of autocorrelation	90th perc	Continuity of cyclic movements
AUTOCORFREQ	Gyroscope (beyond PA)	5s every 2.5s	Frequency of highest autocorrelation (1.0–3.0Hz)	90th perc	Speed of cyclic movements
MAX5	Gyroscope (beyond PA)	60s every 30s	5 highest vel. peaks	90th perc	Intensity of upper-limb use
STD	Gyroscope (beyond PA)	60s every 30s	Standard deviation of signal	90th perc	Intensity of upper-limb use (by the variance of its movement)
STDMEAN	Gyroscope (beyond PA)	60s every 30s	Standard deviation of signal divided by the mean of the signal	90th perc	Intensity of upper-limb use in relation to maximum capacity (within the 60s scenario)
PEAKRATIO	Gyroscope (beyond PA)	60s every 30s	Ratio of delimited (min. distance to last minimum) and all vel. peaks	90th perc	Movement smoothness (signal-to-noise)
PEAKSPER360	Gyroscope (beyond PA)	60s every 30s	Number of vel. peaks per 360° summed rotation	90th perc	Movement smoothness
MEANPEAK	Gyroscope (beyond PA)	60s every 30s	Average height of vel. peaks	90th perc	Intensity of upper-limb use
PEAKSTD	Gyroscope (beyond PA)	60s every 30s	Standard deviation of the height of vel. peaks	90th perc	Intensity of upper-limb use
PEAKSTDMEAN	Gyroscope (beyond PA)	60s every 30s	Standard deviation of the height of vel. peaks divided by the mean of vel. peaks	90th perc	Intensity of upper-limb use in relation to maximum capacity (within the 60s scenario)
FATREV	Accelerometer (PA)	n.a	Ratio of ≥ 30s and ≥ 5s activity bouts (> 100 milli-g)	Complete dataset	Activity fragmentation (e.g., by fatigability)
RELPA	Accelerometer (PA)	5s every 5s	Relative time with > 100 milli-g	Complete dataset	Physical activity
LOW	Gyroscope (beyond PA)	5s every 2.5s	If > 5 steps/min, ratio of coeff. of autocor. < 0.30 and all > 5 steps/min data points	Complete dataset	Amount of gait with low autocorrelation (e.g., by changes of movement direction)

The dimension was based on our a-priori assumption, with PA standing for physical activity / energy expenditure and beyond PA for sensorimotor capacity

**Accelerometer (incl. pedometer):** Every minute, the number of taken steps, based on the built-in pedometer, was read out. The 95th percentile of the cadence (of all minutewise data points which exceeded 5 steps/min to exclude non-gait intervals) was calculated (**CADENCE95**), as well as the mean of all minutewise pedometer data points exceeding 5steps/min in [steps/min] (**CADENCE**). The coverage of a whole minute allowed to emphasize outdoor walking. Indoors, especially in a room or flat, continuous gait with durations of more than a minute would most probable not take place. The 95th percentile was thought to catch the achieved maximum (excluding outliers, which were expected to occur less frequent than in, e.g., 5s measurement periods; in this sense, the 95th percentile was used instead of the 90th percentile), while the mean also represented fragmentation of distance and activity, i.e., walking, but not continuously, within every included minute.

The 90th percentile of the mean acceleration in [g] (**MEANG**), the mean amplitude deviation (**MAD**; the average deviation from the mean of the signal) in [milli-g], and the taken steps (i.e., movements recognized as steps) per second were read out and the frequency of steps (**STEPFREQ**) was derived, based on consecutive measurements of 5s. The shorter interval for gait assessment was thought to emphasize covering short distances (going to the bathroom or from the patient room to the cafeteria) and therefore potentially a different aspect of gait than the cadence parameters (which were covering 1 full minute).

**Gyroscope:** Every 5s, the mean angular velocity in [°/s] was calculated and the 90th percentile was computed (**ACTI**). This parameter was thought as the gyroscope-equivalent to **MEANG**.

Every 2.5s for the past 5s, the highest coefficient of autocorrelation (**AUTOCOR**) and its frequency in [Hz] in a band of 1.0–3.0Hz were calculated (**AUTOCORFREQ**). This was thought to catch cyclic movements, for instance gait. The relatively short time windows (i.e., 5s) of assessment was chosen to enable to also catch short movement durations (for instance, washing hands or walking indoors).

Every 30s, the last 60s of angular velocity data were read out and a set of kinematic parameters was derived. For each described parameter, the 90th percentile (i.e., reproducible best performance) was computed. The five highest velocity peaks in [°/s] (**MAX5**), the standard deviation of the velocity in [°/s] (**STD**), the standard deviation divided by the mean of the velocity (**STDMEAN**), the ratio of velocity peaks that exceed the last minimum by 0.17°/s (in order to exclude peaks by “human” noise; sensor noise was cancelled by signal smoothing) and all velocity peaks (**PEAKRATIO**; the ratio of intended peaks and the sum of intended and unintended peaks as an estimate of movement smoothness), the number of velocity peaks per 360° (**PEAKSPER360**, in contrast to the other parameters, high values represent the worst performance; as an estimate of movement smoothness [15]), the mean of the velocity peaks [°/s] (**MEANPEAK**), the standard deviation of the velocity peaks [°/s] (**PEAKSTD**), and the standard deviation of the velocity peaks divided by the mean (**PEAKSTDMEAN**).

**Summary parameters:** For the full recording, the ratio of activity bouts (MAD > 100 milli-g) of at least 30s and of at least 5s was computed (**FATREV**; accelerometer, higher values indicate lower fragmentation). Further, the relative amount of time of at least light physical activity (MAD > 100 milli-g) (**RELPA**; accelerometer), and the amount of gait with low autocorrelation (at least 5 recognized steps/min and a coefficient of correlation below 0.30) (**LOW;** gyroscope) were derived.

A summary of all used parameters is given in Table 2. Based on a-priori assumptions, we assigned dimensions, i.e., PA and beyond PA, to the parameters. PA means that we assumed a parameter to be closely related to energy expenditure. This included the gait parameters (CADENCE95, CADENCE, STEPFREQ), parameters describing average acceleration (MEANG) and the average of its fluctuation (MAD), and parameters that are based on volume (RELPA) and fragmentation (FATREV) of energy expenditure. We assumed all gyroscope parameters to rather describe the quality than the intensity or volume of behavior, since they mostly rely on wrist rotations that move only small masses (the hand and everyday objects like a pen or glass of water)—and even rotations of the whole upper-limb (by turning of the shoulder) are expected to rotate along the axis of shoulder and hand and in direction of gravity when executing tasks with higher torque demands. The variance in these measures is therefore rather connected to strength, dexterity, and potentially personality (speed-accuracy trade-off) than the cardiovascular capacity and physiological workload. Further, we expected the gyroscope derived parameters to be driven by (rotational) upper-limb, especially hand, movements that are not made to move larger masses like the own body mass during gait (leading to meaningful energy expenditure). Still, rotational movements can be more or less intense. This can be very well described by peak-based metrics. Such metrics extract local maxima, ignoring phases of inactivity and only taking actions into account. MEANPEAK would describe the average reached angular velocity of movements, for instance, when opening a lid or combing one’s hair. Higher values would indicate that these movements were carried out quicker (with higher peak velocities), meaning more intense. Deriving the standard deviation of the reached peaks (PEAKSTD) would further describe to what extent one can adapt the movement intensity—scooping a hot soup should not necessarily be too intense. However, higher values (i.e., variance) can only be achieved if higher intensities are reached (the lower limit of zero cannot be shifted), so PEAKSTD indicates the intensity of movements, but is also taking into account that the intensity should be adapted (to a task). In order to extent this thought, we also derived the quotient of standard deviation and the average (PEAKSTDMEAN), examining the adaptability within the range of one’s capacity to carry out intense movements.

### Statistical analysis

In a first step, parameters with the highest number of strong correlations with all other parameters were identified and used as anchors (*starting points*) for a principal component analysis (PCA; forward selection by Kaiser–Meyer–Olkin criterion). By that, a set of parameters was built, with a measure of sample adequacy (MSA) of at least 0.65 per parameter (Kaiser–Meyer–Olkin criterion) and minimum communalities of 0.65. The number of components was derived from a scree plot. The component scores were then fed into a medoid-based cluster analysis, with the number of clusters being drawn from a scree plot. The clusters where then compared in their clinical and behavioral properties using analyses of variance. Further, the strongest and most unidimensional TUM:W parameters were taken (for each resulting component of the PCA) and examined concerning their association with the NAL measures and models of multiple linear regressions were computed for parameters of upper-limb sensorimotor capacity (i.e., GRIP, PEG, TAP, and DOT) by the respective TUM:W parameter and the EDSS to estimate the specificity of association (general constitution vs. upper-limb capacity). All analyses were run using Rstudio (R version 4.0.5, Rstudio version 1.4.1106, Rstudio PBC, Boston, MA). Parameter distributions were examined for normality using qq-plots and in case of PEG normal distribution was achieved by using the reciprocal of the trial durations. The thresholds for strengths of associations were set to: weak effects r ≥|0.10|, moderate effects r ≥|0.30|, and strong effects r ≥|0.50|. α was set to 0.05.

## Results

Of the recruited 91 participants, 90 watch datasets were used [1 (1%) was excluded due to a relapse, 0 (0%) datasets were missing], 76 had complete datasets [all tests of the NAL test battery, i.e., 14 (16%) missing at least one lab test]. The 76 datasets (Table 1) were used for the PCA and clustering, excluding persons with an inability to perform all lab tests, including walking and standing freely (with closed eyes), and in two cases showing such little activity that FATREV could not be calculated (the respective EDSS were 5.0 and 6.0 and RELPA 1% and 3%). The average wear time (which corresponded to battery time) was 350 ± 87 min (245–541 min). Table 3 gives an overview of the key parameters and their descriptive statistics.

**Table 3:** Listing of key parameters and their descriptive statistics of the full sample (n = 90)

Parameter	Descriptives	Operationalization	Parameter	Descriptives	Operationalization
TAP in [Hz]	12.0 ± 2.1 (7.1–18.1)	Sum of both hands: upper-limb tap frequency (10s)	NORM10 in [s]	12.5 ± 24.5 (3.6–217)	Time to cover 10m, self-paced
GRIP in [kg]	Male: 82.1 ± 20.2 (49.6–125.8) Female: 47.6 ± 10.8 (22.0–72.8)	Sum of both hands: grip strength	MAX10 in [s]	12.5 ± 24.2 (5.7–217)	Time to cover 10m, maximum speed
DOT in [mm]	14.5 ± 2.5 (9.8–23.4)	Sum of both hands: Average distance in a tracking task	TSUG in [s]	13.6 ± 19.8 (1.4–146)	Time to perform the timed stand up and go test (2 × 3m)
PEG in [1/s]	0.021 ± 0.006 (0.006–0.032)	1/sum of both hands: Trial duration in the nine-hole peg test	FREQ in [Hz]	1.75 ± 0.35 (0.0–2.2)	Step frequency during self-paced gait
RT in [ms]	500 ± 76 (374–754)	Mean simple reaction time (10 trials)	COMPLEXITY	0.60 ± 0.23 (0.11–0.93)	Power spectrum derived smoothness of gait, self-paced
GNG in [ms]	596 ± 94 (449–866)	Mean go/no go reaction time (10 trials)	NOISE	0.69 ± 0.16 (0.18–0.91)	Low-pass filter derived signal-to-noise ratio gait, self-paced
SWAY in [mm/s^2^]	85 ± 23 (43–154)	Mean acceleration of waistline during parallel stance (10s)	ASYM	0.23 ± 0.24 (0.01–0.97)	Power spectrum derived asynchronicity of gait, self-paced
ROMBERG	1.41 ± 0.82 (0.85–7.05)	Mean acceleration of waistline during parallel stance with closed eyes (10s)	LIMP	0.09 ± 0.05 (0.02–0.28)	Acceleration derived limping during self-paced gait
LAT	0.09 ± 0.06 (0.02–0.37)	Laterality index of lab tests (NAL)	STD in [°/s]	62 ± 12 (32–107)	Standard deviation of angular vel
CADENCE95 in [1/min]	92 ± 24 (29–160)	Steps/min	STDMEAN	2.19 ± 0.35 (1.48–3.60)	Standard deviation of signal divided by the mean of angular vel
CADENCEin [1/min]	44 ± 13 (18–89)	Steps/min if > 5 recognized steps	PEAKRATIO	0.42 ± 0.08 (0.21–0.71)	Signal-to-noise ratio of angular vel. peaks
MEANG in [g]	0.06 ± 0.03 (0.004–0.17)	Mean acceleration	PEAKSPER360 in [1/360°]	8.8 ± 1.3 (6.6–12.7)	Number of angular vel. peaks/360°
MAD in [mg]	153 ± 52 (42–299)	Fluctuation of acceleration	MEANPEAK in [°/s]	142 ± 27 (80–235)	Average height of angular vel. peaks
STEPFREQ in [Hz]	1.76 ± 0.18 (1.24–2.14)	Steps/s	PEAKSTD in [°/s]	84 ± 14 (43–144)	Standard deviation of the height of angular vel. peaks
ACTI in [°/s]	93 ± 27 (35–186)	Mean angular vel	PEAKSTDMEAN	0.85 ± 0.07 (0.68–1.03)	Standard deviation of the height of vel. peaks divided by the mean of vel. peaks
AUTOCOR	0.44 ± 0.03 (0.37–0.57)	Coefficient of autocorrelation	FATREV	0.53 ± 0.16 (0.10–0.83)	Fragmentation of PA
AUTOCORFREQ in [Hz]	2.5 ± 0.1 (2.3–2.6)	Frequency of autocorrelation (1.0–3.0Hz)	RELPA	0.19 ± 0.09 (0.006–0.426)	Relative time with > 100 milli-g
MAX5 in [°/s]	297 ± 59 (150–539)	Maximum of angular vel	LOW	0.52 ± 0.15 (0.10–0.83)	Amount of gait with low autocorrelation

**Fig. 2:**
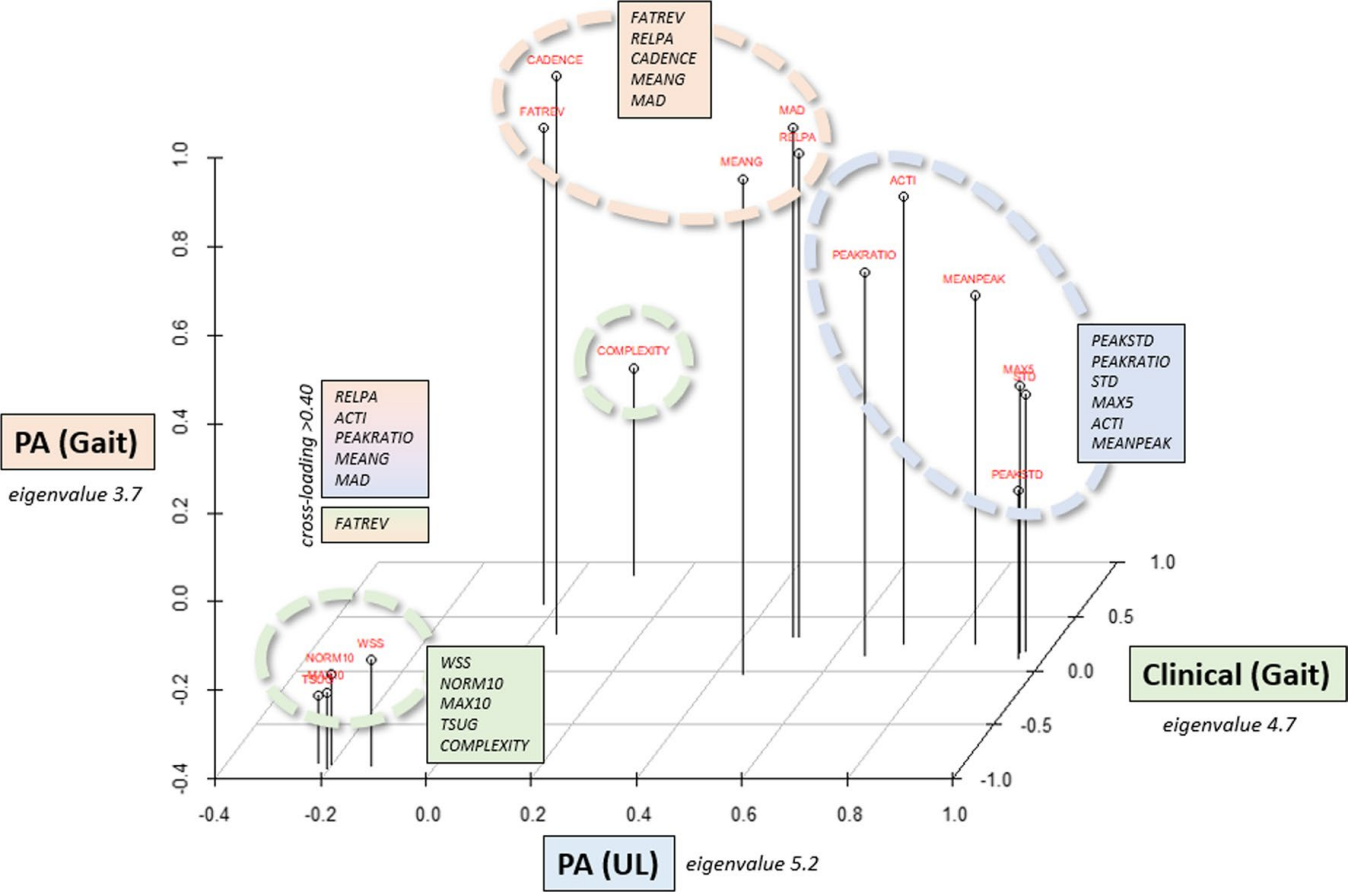
Resulting PCA with 16 parameters and 86.2% explained variance. Components are color coded and circled. Meaningful cross-loadings are indicated by color gradients. In contrast to the other parameters loading on Clinical (Gait), higher values in COMPLEXITY represent better performance. **FATREV**: activity fragmentation, **RELPA**: relative time being physical active, **CADENCE**: walking fragmentation, **MEANG** (mean acceleration): energy expenditure, **MAD** (mean amplitude deviation): energy expenditure, **PEAKSTD** (STD of ang. vel. peaks): movement intensity, **PEAKRATIO** (signal to noise ratio of ang. vel. peaks): movement smoothness, **STD** (STD of ang. vel.): movement intensity, **MAX5** (5 highest ang. vel. peaks): intensity, **ACTI** (mean ang. vel.): intensity, **MEANPEAK** (mean of ang. vel. peaks): intensity, **WSS** (Watzmann Severity Scale): severity of MS, **NORM10** (self-paced time to cover 10m): walking ability, **MAX10** (time to cover 10m at maximum pace): walking ability, **TSUG** (timed stand up and go): functional gait capacity, **COMPLEXITY** (quality of gait): walking ability

**PCA:** The PCA resulted in three components (eigenvalues of 5.2, 4.7, and 3.7; 86.2% explained variance). The MSA was 0.86, ranging from 0.77 to 0.93 and the communality was 0.86 (0.66–0.97), indicating a successful selection of a total of 16 parameters (i.e., the PCA was based on 16 parameters). Based on the loading components, we labeled the components **PA (UL)** (physical activity with an emphasis on upper-limb; we did not choose to label it upper-limb capacity, since there was no loading of upper-limb NAL assessments), **PA (Gait)** (physical activity with an emphasis on gait), and **Clinical (Gait)** (clinical measures with an emphasis on gait capacity) (Fig. 2). Positive component scores indicated better performance, higher activity, or better clinical status, respectively. Although there were cross-loadings of PA parameters, the PCA was overall able to differentiate between **PA (UL)** and **PA (Gait)**. Parameters that were derived from angular velocity were predominantly loading on **PA (UL)**, accelerometer derived parameters on **PA (Gait)**, and gait function from the assessment battery on **Clinical (Gait)**. FATREV revealed a strong cross-loading on the clinical component and had moderate-to-strong associations with the EDSS (r = − 0.58, p < 0.01) and WSS (r = − 0.63, p < 0.01) in single regressions. The most unidimensional parameters were PEAKSTD (**PA (UL)**), COMPLEXITY (**Clinical (Gait)**), and CADENCE (**PA (Gait)**).

**Fig. 3:**
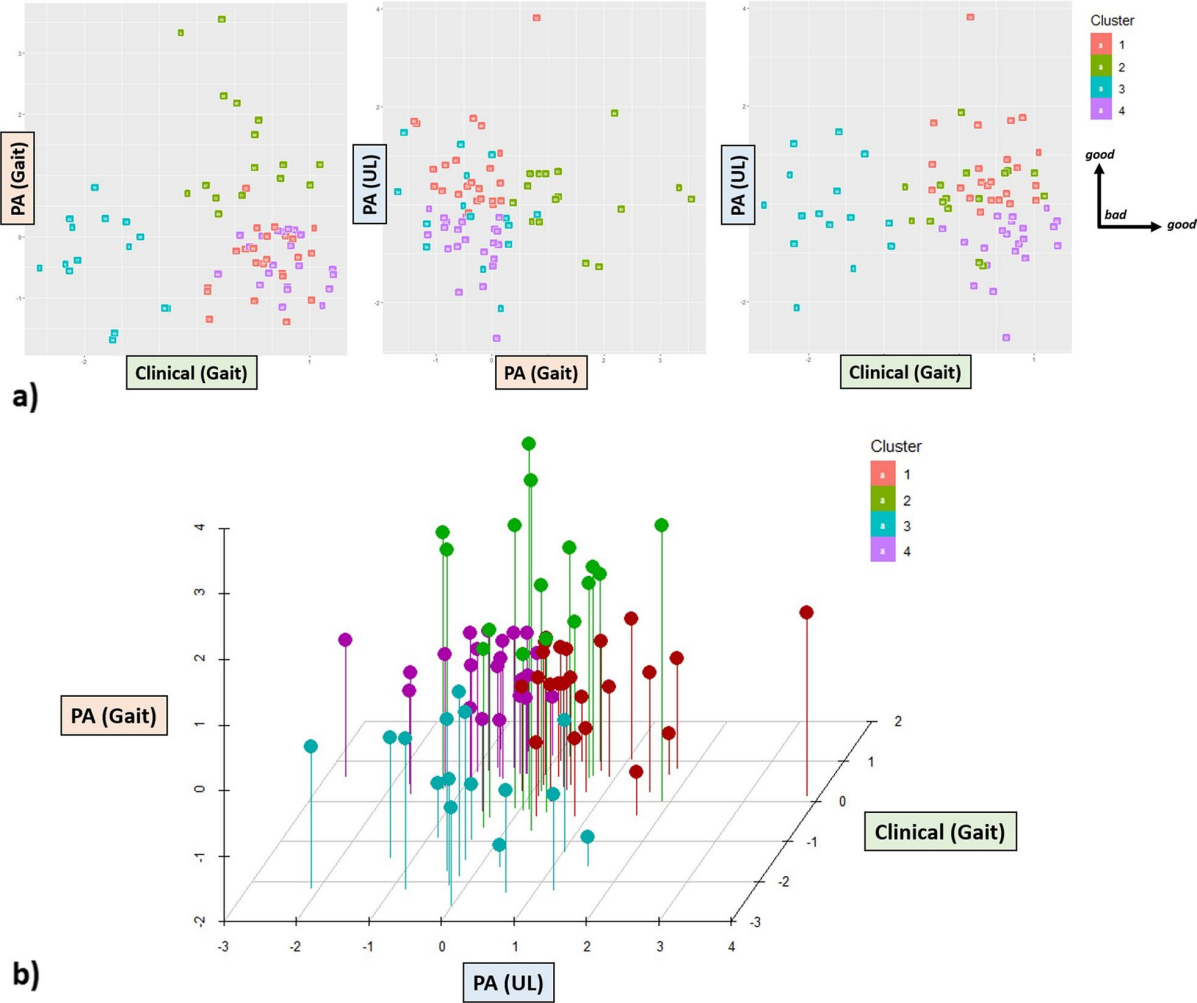
**a** Individual component loadings of the four derived clusters. Lower loadings indicate worse performance in the respective dimension. **b** 3D scatterplot of the individual component loadings of the four derived clusters. Lower loadings indicate worse performance in the respective dimension

**Cluster:** The cluster analysis suggested the best fit with four clusters (Fig. 3). The clusters differed in all three dimensions (all ps < 0.01). Cluster 1 was physically active (upper-limb) (component scores: 0.76 ± 0.89), with a good clinical status (0.41 ± 0.42), and little gait activity (− 0.39 ± 0.53). Cluster 2 revealed an average clinical status (0.12 ± 0.54), average physical activity (upper-limb) (0.15 ± 0.75) and the highest gait activity (1.47 ± 0.96). Cluster 3 was physically slightly inactive (upper-limb) (− 0.14 ± 0.96), had a more severe clinical status (− 1.75 ± 0.50), and very little gait activity (− 0.39 ± 0.74). Cluster 4 was physical inactive (upper-limb) (− 0.81 ± 0.62), had a good clinical status (0.68 ± 0.39), and revealed little gait activity (− 0.39 ± 0.74).

**Fig. 4:**
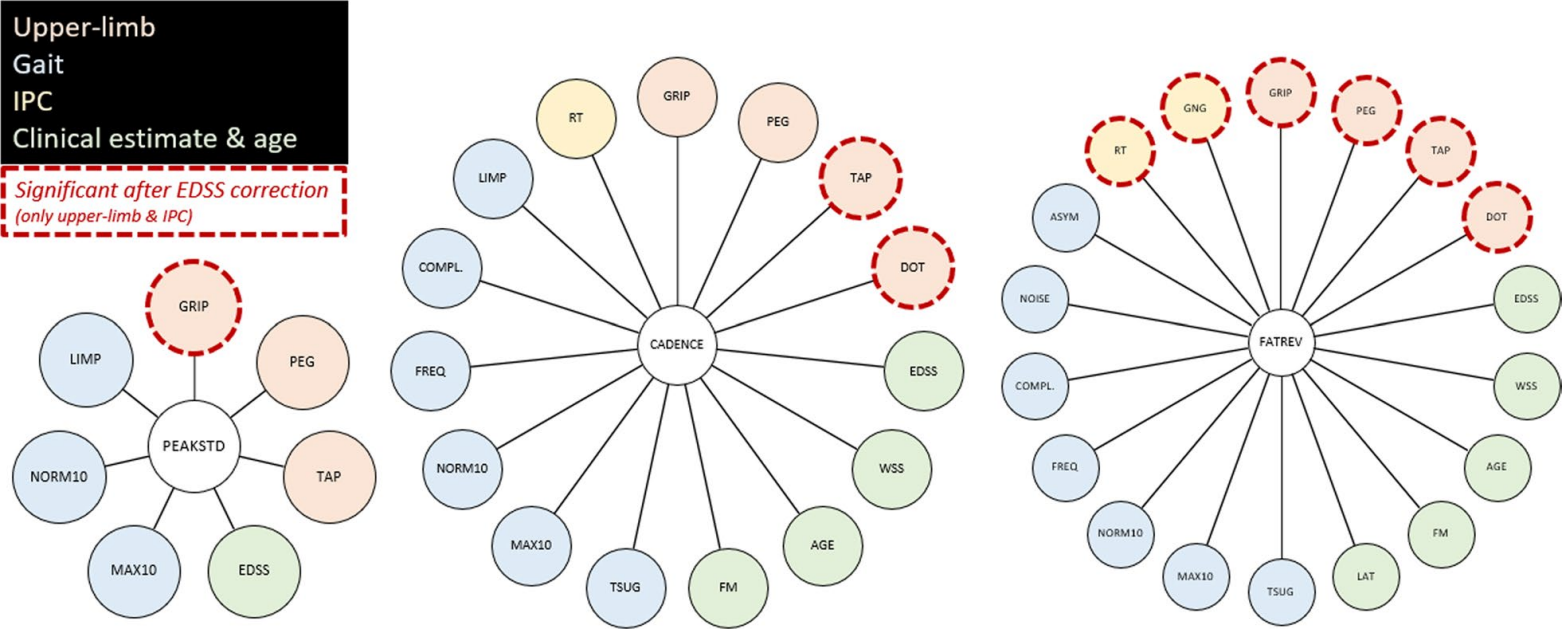
Associations between three key-parameters and performance at the Neuro Assessment Lab, including patient characteristics (green). IPC: Information processing capacity. **PEAKSTD** (STD of ang. vel. peaks): movement intensity, **CADENCE**: walking fragmentation, **FATREV**: activity fragmentation

**Fig. 5:**
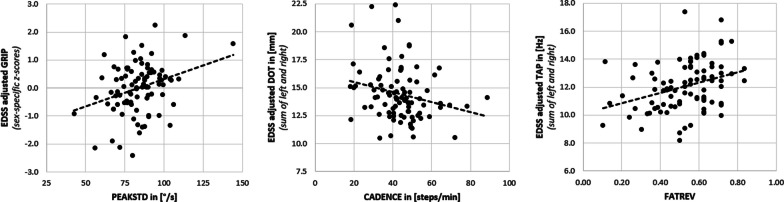
Exemplary scatterplots of PEAKSTD, CADENCE, and FATREV with EDSS adjusted test scores of upper-limb capacity from the NAL. **GRIP** refers to grip strength (higher values indicate better performance), **DOT** to the distance in a manual pursuit task (lower values indicate better performance), **TAP** to the upper-limb tapping frequency (higher values indicate better performance). **PEAKSTD** is the standard deviation of the angular velocity peaks assessed by the smartwatches (higher values indicate higher intensities of upper-limb use), **CADENCE** is the average of recorded cadences in case of gait (higher values indicate less walking fragmentation), **FATREV** is the reverse of activity fragmentation (higher values indicate less fragmentation)

Three TUM:W parameters of interest were further examined (most unidimensional parameters of the three components). PEAKSTD to better understand **PA (UL)**, CADENCE for **PA (Gait)**, and FATREV with its strong cross-loading (0.62) on **Clinical (Gait)** (Table 4, Fig. 4). Figure 5 gives exemplary scatterplots of these three parameters with EDSS adjusted test scores of upper-limb capacity from the NAL.

**Table 4:** Univariate and EDSS adjusted associations between key-parameters and performance at the Neuro Assessment Lab, including patient characteristics

Parameter	Dimension	Univariate regression	EDSS adjusted
PEAKSTD	Upper-limb	GRIP r = 0.44, p < 0.01 PEG r = 0.25, p = 0.028 TAP r = 0.25, p = 0.029	GRIP β = 0.33, p < 0.01
	Gait	LIMP r = − 0.24, p = 0.040 NORM10 r = − 0.27, p = 0.019 MAX10 r = − 0.29, p = 0.010	–
	Information processing capacity	–	–
	Clinical estimate & age	EDSS r = − 0.31, p < 0.01	–
CADENCE	Upper-limb	GRIP r = 0.38, p < 0.01 PEG r = 0.43, p < 0.01 TAP r = 0.44, p < 0.01 DOT r = − 0.40, p < 0.01	TAP β = 0.25, p = 0.042 DOT β = − 0.40, p < 0.01
	Gait	LIMP r = − 0.25, p = 0.032 COMPL r = 0.35, p < 0.01 FREQ r = 0.55, p < 0.01 NORM10 r = − 0.47, p < 0.01 MAX10 r = − 0.50, p < 0.01 TSUG r = − 0.51, p < 0.01	–
	Information processing capacity	RT r = − 0.24, p = 0.034	–
	Clinical estimate and age	EDSS r = − 0.57, p < 0.01 WSS r = − 0.43, p < 0.01 AGE r = − 0.40, p < 0.01 FM r = − 0.34, p < 0.01	–
FATREV	Upper-limb	GRIP r = 0.32, p < 0.01 PEG r = 0.50, p < 0.01 TAP r = 0.57, p < 0.01 DOT r = − 0.55, p < 0.01	GRIP β = 0.57, p < 0.01 PEG β = 0.24, p = 0.034 TAP β = 0.57, p < 0.01 DOT β = − 0.55, p < 0.01
	Gait	ASYM r = − 0.24, p = 0.038 NOISE r = 0.37, p < 0.01 COMPL r = 0.56, p < 0.01 FREQ r = 0.63, p < .01 NORM10 r = − 0.62, p < 0.01 MAX10 r = − 0.67, p < 0.01 TSUG r = − 0.69, p < .01	–
	Information processing capacity	RT r = − 0.37, p < 0.01 GNG r = − 0.27, p = 0.018	RT β = − 0.37, p < 0.01 GNG β = − 0.27, p = 0.018
	Clinical estimate and age	EDSS r = − 0.58, p < 0.01 WSS r = − 0.63, p < 0.01 AGE r = − 0.36, p < 0.01 FM r = − 0.35, p < 0.01 LAT r = − 0.24, p = 0.034	–

“EDSS adjusted” lists parameters that can be predicted by the respective TUM:W parameter with a model including the EDSS

**PEAKSTD**: standard deviation of angular velocity peaks, higher values indicate higher intensities of upper-limb use, **CADENCE**: average steps/min in case of gait, higher values indicate less walking fragmentation, **PEG**: 1/trial duration, higher values indicate better performance; **FATREV**: bouts > 30s/> 5s, higher values indicate lower fragmentation

PEAKSTD was moderately associated with GRIP (r = 0.44, p < 0.01, n = 90) and weakly with PEG (r = 0.25, p = 0.028, n = 90) and TAP (r = 0.25, p = 0.029, n = 90). Further, PEAKSTD was associated with EDSS (r = − 0.31, p = 0.007, n = 90), MAX10 (r = − 0.29, p = 0.010, n = 85), NORM10 (r = − 0.27, p = 0.019, n = 85), and LIMP (r = − 0.24, p = 0.040, n = 82). GRIP was predictable (R^2^_adj_ = 0.27, p < 0.01, n = 90) by PEAKSTD (β = 0.33, VIF = 1.11, p < 0.01) and EDSS (β = 0.33, VIF = 1.11, p < 0.01), while models for PEG and TAP showed no significant impact of PEAKSTD if the EDSS was included.

CADENCE was associated with almost all characteristics and NAL parameters: AGE (r = − 0.40, p < 0.01, n = 90), FM (r = − 0.34, p < 0.01, n = 90), EDSS (r = − 0.57, p < 0.01, n = 90), WSS (r = − 0.43, p < 0.01, n = 90), PEG (r = 0.43, p < 0.01, n = 90), GRIP (r = 0.38, p < 0.01, n = 90), TAP (r = 0.44, p < 0.01, n = 90), DOT (r = − 0.40, p < 0.01, n = 90), RT (r = − 0.24, p = 0.034, n = 90), TSUG (r = − 0.51, p < 0.01, n = 85), MAX10 (r = − 0.50, p < 0.01, n = 85), NORM10 (r = − 0.47, p < 0.01, n = 85), FREQ (r = 0.55, p < 0.01, n = 84), COMPLEXITY (r = 0.35, p < 0.01, n = 84), and LIMP (r = − 0.25, p = 0.032, n = 82). TAP was predictable by CADENCE and EDSS (R^2^_adj_ = 0.25, p < 0.01, n = 90; CADENCE β = 0.25, p = 0.042; EDSS β = − 0.33, p < 0.01; VIF = 1.48) and DOT only by CADENCE (R^2^ = 0.15, p < 0.01, n = 90; CADENCE β = − 0.40, p < 0.01).

FATREV also revealed a plethora of significant correlations. It was associated with AGE (r = − 0.36, p < 0.01, n = 88), FM (r = − 0.35, p < 0.01, n = 88), EDSS (r = − 0.58, p < 0.01, n = 88), WSS (r = − 0.63, p < 0.01, n = 88), PEG (r = 0.50, p < 0.01, n = 88), GRIP (r = 0.32, p < 0.01, n = 88), TAP (r = 0.57, p < 0.01, n = 88), DOT (r = -0.55, p < 0.01, n = 88), RT (r = − 0.37, p < 0.01, n = 88), GNG (r = − 0.27, p = 0.018, n = 88), TSUG (r = − 0.69, p < 0.01, n = 83), MAX10 (r = − 0.67, p < 0.01, n = 83), NORM10 (r = − 0.62, p < 0.01, n = 83), FREQ (r = 0.63, p < 0.01, n = 82), COMPLEXITY (r = 0.56, p < 0.01, n = 82), NOISE (r = 0.37, p < 0.01, n = 81), ASYM (r = − 0.24, p = 0.038, n = 82), and LAT (r = − 0.24, p = 0.034, n = 88). The associations with AGE, WSS, GRIP, TAP, DOT, RT, GNG, and FREQ were stronger than of the EDSS. PEG was predictable (R^2^_adj_ = 0.37, p < 0.01, n = 88) by FATREV (β = 0.24, VIF = 1.50, p = 0.034) and EDSS (β = − 0.45, VIF = 1.50, p < 0.01). GRIP was predictable by FATREV (R^2^ = 0.32, p < 0.01, n = 88; FATREV β = 0.57, p < 0.01), but not EDSS, same as DOT (R^2^ = 0.31, p < 0.01, n = 88; FATREV β = − 0.55, p < 0.01), TAP (R^2^ = 0.31, p < 0.01, n = 88; FATREV β = 0.57), RT (R^2^ = 0.14, p < 0.01, n = 88; FATREV β = -0.37, p < 0.01), and GNG (R^2^ = 0.07, p = 0.018, n = 88; FATREV β = − 0.27, p = 0.018).

## Discussion

In this exploratory, cross-sectional study, we examined the physical behavior of 90 pwMS in an inpatient neurorehabilitation setting. Our goal was to go beyond the sheer estimate of energy expenditure by using a set of kinematic parameters derived from the sensors of a smartwatch. Our analyses suggested that two distinct dimensions of PA were assessed. One being gait related—increased energy expenditure by gait—and one being upper-limb determined—increased energy expenditure by everyday hand use. Further, based on a lab assessment of sensorimotor control (upper-limbs, information processing capacity, gait) and estimates of the clinical severity of the MS (i.e., EDSS and WSS), a third component was derived. This, however, did not include kinematic parameters from the smartwatch and had a strong emphasis on gait. In a third analytical step, we examined the potential to describe sensorimotor capacity by TUM:W parameters—to go beyond PA.

### PA clusters

Patients revealed different behavioral stereotypes (as previously reported for self-reported fatigue [19], that strongly differed in the amplitude of upper-limb PA, gait-related PA, as well as their clinical status. Cluster 1 revealed greater amounts of upper-limb PA, while showing little gait PA, with an overall good clinical status. Cluster 2 showed average upper-limb PA and clinical status, but a high level of gait PA. Cluster 3 was slightly inactive (upper-limb PA), showed little gait activity, and had the most severe clinical status. Cluster 4, although having a good clinical status, showed little gait activity and the least upper-limb PA. Cluster 3 showed an expectable behavior concerning the clinical status (i.e., more severe clinical status, physically slightly inactive, and very little gait activity). Cluster 1 and 2 suggest that there could be different emphases of PA, either trying to maximize ambulatory activity or upper-limb movements, although it remains unanswered how these upper-limb PA levels were achieved—by ADL, upper-limb trainings, gesticulating while conversing, or recreational activities (knitting, gaming, nose-picking, etc.). Although cluster 4 had the best clinical status, they revealed little to very little PA of upper-limbs and gait. These could be indicators of sedentary behavior, potentially caused by psychological factors like experienced fatigue, depression, or low motivation; it has been previously reported that higher levels of experienced fatigue are rather accompanied by lower levels of PA than activity fragmentation, which would be the case for cluster 4 (FATREV [fragmentation of PA] cluster 4 = 0.56 ± 0.08 vs. FATREV all participants = 0.54 ± 0.15, p = 0.642) [17, 30]. Taken together, the clusters show that (a) just because one can walk doesn’t mean that one will walk, (b) the amount and intensity of usage of upper-limbs is quite independent from the clinical status, and (c) that sedentarism might be limb-specific, i.e., persons with a lot of gait activity do not necessarily show a lot of upper-limb activity. It will be necessary to examine the impact of personal interests, therapy content, intensity, applied therapists, medication, as well as cognitive and emotional status on the translation of clinical status (e.g., the ability to walk fast or use the hands in a dexterous way) into everyday behavior.

### Going beyond PA

We explored three TUM:W parameters of particular interest; parameters with strong unidimensional loading for each of the extracted components. Each of the extracted three components represented a certain aspect of capacity and/or everyday behavior. If being able to gather information of sensorimotor capacity (e.g., grip strength, finger dexterity, or gait quality) from wearable-derived parameters, it would allow us to shift from a lab-based scenario with discrete measurement points to a continuous assessment strategy. This could also cover fluctuations, the course of therapy or disease, and provide the opportunity to go beyond PA, for instance, distinguishing between a sedentary healthy person and a physically active person with a neurological disease, or early detecting persons at risk/without diagnosis. Beyond PA, in this context, is the estimation of sensorimotor capacity, independent of physical activity.

PEAKSTD [standard deviation of angular velocity peaks] showed connections with gait and upper-limb performance and the EDSS (Figs. 4, 5). When examining the sensorimotor capacity of the upper-limbs, only GRIP was not fully mediated by the EDSS. In this respect, the standard deviation of the angular velocity peaks was a moderate predictor of sex specific grip and—considering the gait speed associations—general physical strength (as grip strength has been shown to estimate general physical strength in clinical populations [34]. Increased physical strength would allow an individual to fully adapt its actions to the demands of different actions (e.g., slow manipulation vs. quick transport), while weaker individuals tend to rather show monotonous behavioral patterns due to their limited task-adaptability as it has been shown for elderly with higher levels of frailty [32]. It is important to note that the parameter was based on angular velocity, i.e., rotation and not translation of the wrist was the driving factor. The ability to predict the sex-adjusted grip-strength makes PEAKSTD a parameter with great potential for populations with a (pathological) loss of muscle mass and/or function (e.g., frail elderly), not only to assess the progression, but also to observe rehabilitation outcomes in an externally valid way (actual use in everyday life).

The derived mean cadence (CADENCE) of recognized minute wise ambulatory activity was associated with almost all lab assessments (Fig. 4), covering the full range of upper-limb capacity, gait performance, as well as clinical estimates. The strong association with the calendar age of patients, stronger than the correlation with FM [time since first manifestation of MS], indicates that age should always be concerned, independent of neurological health status [27]. CADENCE was a good predictor of the global condition of a subject, but very unspecific (i.e., CADENCE was a good predictor of a multitude of parameters,see Table 4, Figs. 4, 5). One can derive the status or the changes of status without knowing the underlying mechanisms. Gait is frequently called the “sixth vital sign” [11] and is, as in our case, often a strong, but unspecific symptom [29, 35]. Additionally, cluster 4 indicated that gait-related PA alone can be misleading, as it had a good clinical status (with an emphasis on gait capacity) but revealed only little gait. Walking little can stem from an impaired gait function, a low motivation to walk, or a combination of both. Further, one should keep in mind that a low fitness level has been shown to be a risk factor for developing MS [6]. Cortese et al. [6] (successfully) used the duration for a 3000m run of 19a men (Norwegian conscription measure) to estimate the impact of aerobic fitness on the prevalence of MS with onsets later than 10a after the fitness test, therefore controlling for potential confounding early disease signs. In this sense, i.e., low fitness levels being a risk factor for MS, CADENCE alone might not be the best measure to go beyond PA, although adding the percentile-based metric of the step frequency or cadence to the currently used daily step counts [1, 4, 5, 17, 20] might be a valuable addition (not only in neurological samples). The remaining association of CADENCE and TAP [upper-limb tapping] after controlling for the EDSS indicates that the average cadence is already deteriorating with decreasing conductivity (tapping tasks have been shown to be markers of reduced central nerve conductivity [20] before reduced gait distances become apparent in the EDSS scoring system. The connection of CADENCE with DOT [pursuing a moving target with the finger] (approx. 15% shared variance) rather underlines the missing representation of non-self-paced time pressure tasks in the disease rating (DOT have been shown to be moderately associated with the EDSS [18], although the multiple sclerosis functional composite used to include the paced auditory serial addition task [26]. Interestingly, but in accordance with [36], fast lab walkers were apparently not automatically fast everyday walkers, as there was no connection between gait velocity (in the lab) and CADENCE after controlling for the EDSS. One potential alternative explanation for the connection of CADENCE with TAP and DOT would be that strong upper-limb movements have been wrongly recognized as steps by the pedometer and persons with better upper-limb capacity would show more misrecognizable activity. However, a missing cross-loading on **PA (UL)** (Fig. 2) speaks against this.

FATREV [fragmentation of PA], the inverse ratio of short and long activity bouts, with higher values indicating less fragmentation, revealed, like CADENCE, a very global connection to the almost the complete test battery of the NAL (Figs. 4, 5). Same as CADENCE, it appeared to be comprehensive but unspecific. A deeper examination, however, showed that FATREV was well able to go beyond PA (after controlling for EDSS mediation), being a moderate to good predictor of upper-limb capacity and even weakly of information processing capacity. FATREV (EDSS adjusted) was able to predict the (inverse) nine hole peg test performance, grip strength, upper-limb tapping, pursuit under time pressure, and reaction times (simple and go/nogo). Especially the grip strength, tapping, and pursuit task predictions had quite strong |β|-weights of > 0.50. Studies from ageing and stroke have revealed for complex ADL that increased fragmentation (active times during task execution, usually referred to as *relative activity*) during execution is a good predictor of overall performance (i.e., trial duration) in elderly [13, 16], further that performance in neurological populations (i.e., stroke survivors) could be globally reduced (i.e., independent of sub-action demands) [12], and that higher individual demands of a tasks can also lower the execution speed and therefore sub-pass the threshold of ‘activity’ (i.e., activity being meaningful for increasing the energy expenditure) for wearables [16, 32]. Too what extend fatigability [19, 30], cognitive task demands [12, 13], or limited sensorimotor capacity is leading to a reduced FATREV, however, stands to be answered in future studies, as we did not include tasks on fatigability (motor/&cognitive in the NAL test battery).

The potential advantage of the three discussed parameters in comparison to conventionally assessed capacity measures is that they can be derived from wearables and therefore be used in a continuous way (see discussion above). Further, assessing persons in their home environment (including the nearby community), would allow us to have externally valid information of sensorimotor capacity (and its fluctuations and changes), as an additional dimension to physical activity measures.

The use of the 90th percentile was aiming at assessing the best possible performance during daily life without being too prone to outliers or measurement errors. In future it would be of interest to see if such derived values tend to be stable over longer measurement durations. Further, gyroscopic data appeared to be better suited to go beyond PA, since there appeared to be less “cross-talk” with gait, like it is often observed in pure accelerometric assessments [7, 23, 24], and due to the fact that rotation (and orientation) of the forearm is crucial for ADL performance [9, 23], however, a direct comparison of comparable gyroscopic and acceleration based parameters is still lacking. Different sensor placements might also allow to assess gait and postural stability in daily life, as there have been promising approaches [10]. The extent of transferability of our findings to world settings (not inpatient rehabilitation), longer measurement periods, or healthy samples will be covered in future studies. So far, battery life remains the strongest barrier in this field of research (without losing information by down-sampling) [7]. Another promising approach would be to examine the impact of therapy volume, exercise intensity, and the executing therapist on behavior and its changes. Such data could be used to optimize and personalize therapeutic measures.

## Conclusion

Although reporting limited explained variance of lab-assessed sensorimotor capacity in a patient sample, we report first prove of feasibility of deriving meaningful information that goes beyond estimated energy expenditure. However, we only used one measurement point, so we have no information on the reliability of the approach. Such assessment, if proven to be reliable, might help to supervise interventions or to early identify persons at risk of losing independence in daily life.

## Data Availability

The dataset supporting the conclusions are available upon reasonable request to the corresponding author.
